# Characteristics and clinical relevance of leukocytic response in children with diabetic ketoacidosis – A comparative cohort study

**DOI:** 10.20945/2359-4292-2023-0183

**Published:** 2024-07-30

**Authors:** Harish Kumar, Bhanu Kiran Bhakhri, Vernika Tyagi, Nupur Singh, Dharmendra Kumar Singh, Ruchi Rai

**Affiliations:** 1 Department of Pediatrics Institute of Child Health Atal Bihari Bajpai Medical University Noida Uttar Pradesh India Department of Pediatrics, Post Graduate Institute of Child Health (affiliated to Atal Bihari Bajpai Medical University, Lucknow), Noida, Uttar Pradesh, India; 2 Department of Neonatology and MRH Institute of Child Health Atal Bihari Bajpai Medical University Noida Uttar Pradesh India Department of Neonatology and MRH, Post Graduate Institute of Child Health (affiliated to Atal Bihari Bajpai Medical University, Lucknow), Noida, Uttar Pradesh, India

**Keywords:** Diabetic ketoacidosis, leukocytosis, cerebral edema, type 1 diabetes mellitus, differential leukocyte count

## Abstract

**Objective:**

Leukocytosis is often observed among children presenting with diabetic ketoacidosis (DKA). This study compares detailed parameters of leukocytosis in children presenting with DKA versus infection.

**Subjects and methods:**

In this comparative cohort study, we collected data from two groups of children, one hospitalized with DKA and another with community-acquired pneumonia (CAP). The primary objective was to compare the neutrophil-to-lymphocyte ratio (NLR) between the groups. Total leukocyte count (TLC), absolute neutrophil count (ANC), platelet-to-lymphocyte ratio (PLR), and platelet-to-monocyte ratio (PMR) were also compared. The correlation of these hematological parameters with the clinical outcomes in the DKA group was also explored.

**Results:**

Data from 35 children with DKA (mean age 7.4 years, 12 boys) and 40 children with CAP (mean age 7.9 years, 15 boys) were available for comparison. No significant NLR difference was observed between the DKA and CAP groups. Similarly, no significant difference was observed in TLC and ANC between the groups. However, significant differences between the DKA and CAP groups were observed regarding mean (standard deviation) PLR (108.26 [67.51] versus 166.60 [163.83], respectively, p = 0.01) and mean PMR (1,795.40 [4,307.00] versus 886.33 [1,726.41], p = 0.01). Among children with DKA, ANC and PMR correlated positively and hemoglobin level correlated negatively with unfavorable outcomes.

**Conclusions:**

Specific parameters of leukocytosis (PLR and PMR) differed significantly in children with DKA versus CAP. Some widely available and inexpensive hematological parameters of inflammation (hemoglobin, ANC, and PMR) may predict outcomes in patients with DKA.

## INTRODUCTION

Diabetic ketoacidosis (DKA) is an acute life-threatening complication of type 1 diabetes mellitus (T1DM) in children. It is the end result of absolute or relative insulin deficiency combined with an excess of counterregulatory hormones (*i.e.*, glucagon, catecholamines, cortisol, and growth hormone). About 25%-40% of the children with newly diagnosed T1DM present with DKA (1), which is usually precipitated by physiological stressful conditions.

Notably, DKA is considered a condition with stimulated immune response, with a number of biochemical inflammatory markers reportedly elevated during its acute phase (2). Several studies have shown that DKA is associated with an inflammatory response and altered hematological parameters (3,4). By itself, DKA mimics infection, and differentiation of septic from nonseptic inflammatory response may be difficult (5). Hence, many patients are overtreated with antibiotics, leading to inadequate treatment costs, side effects, and bacterial resistance.

A leukocytic response is commonly observed during DKA. However, characteristics of this response have never been explored appropriately, especially regarding the differential leukocyte count. It is unclear if the leukocytic response seen among children with DKA is similar to that seen among children with infection. Characteristics of the leukocytic response with the potential to predict some outcomes in the course of the illness have also remained entirely unexplored.

The role of various hematological parameters (*e.g.*, neutrophil-to-lymphocyte ratio [NLR], absolute neutrophil count [ANC], platelet-to-lymphocyte ratio [PLR], and platelet-to-monocyte ratio [PMR]) in predicting outcomes during and after infection have been recently emphasized during the COVID-19 pandemic (6,7). These hematological markers have also been associated with activity in some noninfectious inflammatory conditions (8). The NLR is known to be associated with inflammation and subclinical inflammation in coronary artery disease and certain malignancies (9-13).

Based on these considerations, the purpose of this study was to analyze the various hematological parameters of inflammation (total leukocyte count [TLC], differential blood cell count, NLR, ANC, PLR, and PMR) in children with DKA compared with children with community-acquired pneumonia (CAP), a common infectious condition. The correlation of these parameters with disease outcomes (including duration of hospital stay, development of shock, cerebral edema, and death) was also explored in the DKA group.

## SUBJECTS AND METHODS

This was a comparative cohort study with data collected both retrospectively and prospectively. The study was conducted over a period of 18 months at the emergency care unit of a tertiary care pediatric teaching hospital located in northern India. The retrospective data were collected from children who visited the unit between January 2017 and December 2020 and fulfilled the eligibility criteria. Prospective participants were enrolled between January 2021 and June 2022. Retrospective data collection was performed only for children in the DKA group, while all the participants in the CAP group were enrolled prospectively.

Informed consent was obtained from a parent or guardian of each child invited for prospective participation. The ethical committee of the institute approved the conduct of the study.

### Research question

The study sought to clarify whether hematological parameters of inflammation differ between children with DKA (“case group”) *versus* children with CAP (“control group”) at the time of presentation.

### Eligibility criteria

The inclusion criteria were children aged 1-18 years admitted to the pediatric emergency unit. The inclusion criteria also included a diagnosis of DKA according to the ISPAD 2018 consensus guidelines (14) for the case group and a diagnosis of CAP according to standard guidelines (15) for the control group.

The exclusion criteria for both groups were preexisting chronic illness, current use of any long-term medication, and blood transfusion during the past year. Since the prospective enrollment was done during the period overlapping with the global COVID-19 pandemic, all the children were enrolled after ruling out active COVID-19 infection.

In the DKA group, children with microbiological/radiological evidence of infection (blood or body fluid cultures, chest x-ray, relevant markers measured for common endemic infections like viral hepatitis, dengue, malaria, typhoid fever, rickettsial illnesses, etc.) were excluded.

The primary objective was a comparison between groups regarding NLR in the baseline sample. The secondary objectives were a comparison between groups regarding TLC, ANC, PLR, PMR, and differential white blood cell count, and an exploration between baseline hematological parameters and various clinical characteristics and outcomes among children with DKA.

### Sample size and statistical analysis

A TLC comparison in sick children with and without DKA was used for sample size calculation, as data regarding NLR was not available in the literature. Considering a mean (standard deviation [SD]) TLC value of $18.3(6.6)\times 10^{3}/{mm}^{3}$ in patients with DKA and $14.3(6.1)\times 10^{3}/{mm}^{3}$ in patients with CAP, as reported by Karavanaki and cols. and Çağlayan Serin and cols., respectively, a sample size of 40 was required in each group considering 80% power and 95% confidence level. (16,17) The corresponding figure considering 90% confidence interval was 32 in each group. However, considering the restrictions related to the ongoing COVID-19 pandemic, the plan was to achieve the best possible enrollment.

The data was recorded in Microsoft Excel spreadsheets and analyzed using SPSS (IBM Corp., Armonk, NY, USA) and OpenEpi (www.openepi.com) software. The baseline hematological parameters (hemoglobin, cell counts, ratios between different types of cells, etc.) were compared between both groups. In addition, their correlation with various clinical outcomes (severity of illness, duration of hospital stay, need for vasopressor or ventilator support, complications, and final outcome) was explored. Quantitative data were presented as mean (SD) and compared using Student’s *t* test, and categorical data were presented as proportions and analyzed using the chi-square test. Pearson’s correlation was used to calculate the relationship between the hematological parameters and the clinical outcomes in the DKA group. P values < 0.05 were considered significant.

### Procedure

The data collected at baseline included sociodemographic details and relevant clinical information (symptom type and duration, key examination findings, previous treatment details, etc.). Hematological parameters were measured at baseline as part of the initial blood evaluation and according to clinical requirements. The data collected during the course of the illness included records of clinical progression, severity of illness, laboratory investigation results, duration of hospital stay, need for vasopressor or ventilator support, description of complications, final outcomes, etc.

### Sample collection and analysis

Random blood glucose was measured on capillary blood using the blood glucose monitor AccuSure (MicroGene Diagnostic Systems Ltd, India). Heparinized venous samples were analyzed for blood gas parameters using an automatic analyzing system (Combi Line, Eschweiler GmbH & Co KG, Germany). Blood samples for measurement of hematological parameters were collected in EDTA tubes and processed using a 5-part automated analyzer (CELL-DYN Ruby, Abbott Core Laboratory, Abbot Park, IL, USA). Urinary ketones were analyzed using reagent strips (Keto-Diastix, Siemens Healthcare Pvt. Ltd., India).

## RESULTS

**Figure 1:**
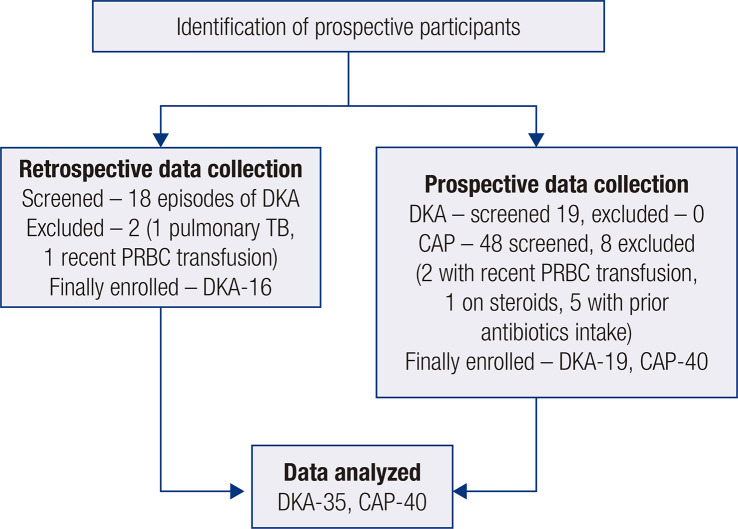
Flow chart of details relevant to the study data collection.

Abbreviations: CAP, community-acquired pneumonia; DKA, diabetic ketoacidosis;PRBC, packed red blood cells; TB, tuberculosis.

Data from 35 children with DKA were available at the end of the study period. Of these, collection of data was done retrospectively in 16 and prospectively in 19. Additionally, 40 children with CAP participated in the study, all of whom were enrolled prospectively. Details on data collection are shown in Figure 1.

The final study sample included 35 children with DKA (12 boys and 23 girls; mean age 7.4 [4.08] years) in the case group and 40 children with CAP (15 boys and 25 girls, mean age 7.9 [3.57] years) in the control group. There was no significant difference in age or sex distribution between the groups. Among the 35 children with DKA, the DKA severity, according to ISPAD 2018 guidelines (14), was graded mild in 8 (22.8%), moderate in 25 (71.4%), and severe in 2 (5%) of them. Among the 40 children with CAP, the pneumonia severity, according to WHO criteria (15), was graded nonsevere in 19 (47.5%), severe in 13 (32.5%), and very severe in 8 (20%) of them.

Children with DKA were significantly more likely to present with abdominal pain (p < 0.05), vomiting (p < 0.01), and dehydration (p < 0.01) compared with those with CAP. In all, 22 of the 35 children with DKA and 1 of the 40 children with CAP were dehydrated at presentation. Symptom duration was longer among children with DKA. No significant differences between groups were observed regarding the incidence of shock requiring vasopressor therapy, respiratory failure requiring mechanical ventilation, and duration of hospital stay.

Table 1 shows a comparison of hematological parameters between the groups.

**Table 1:** Comparison of hematological parameters* between the groups

Parameters	Case group (n = 35)	Control group (n = 40)	P value
Neutrophil-to-lymphocyte ratio	4.77 (3.35)	6.13 (9.36)	0.10
Total leukocyte count (×1,000/mm^3^)	18.78 (9.70)	14.22 (8.55)	0.50
Absolute neutrophil count (×1,000/mm^3^)	14.21 (8.37)	12.10 (14.51)	0.39
Lymphocyte count (×1,000/mm^3^)	3.67 (2.37)	2.8 7 (2.37)	0.66
Platelet count (×1,000/mm^3^)	291.91 (101.72)	275.71 (139.16)	0.45
Platelet-to-lymphocyte ratio	108.26 (67.51)	166.60 (163.83)	**0.01**
Monocyte count (×1,000/mm^3^)	1.22 (1.08)	0.88 (0.92)	0.26
Platelet-to-monocyte ratio	1,795.40 (4,307.00)	886.33 (1,726.41)	**0.01**
Eosinophil count (×1,000/mm^3^)	0.56 (1.41)	0.29 (0.80)	**0.01**
Basophil count (×1,000/mm^3^)	0.17 (0.24)	0.21 (0.31)	0.25
Red blood count (×10^6^/mm^3^)	4.95 (0.72)	4.05 (0.75)	0.98
Hemoglobin (g/dL)	12.41 (2.30)	9.70 (2.02)	0.40
Hematocrit (%)	39.47 (6.08)	30.41 (5.55)	0.69

*Values are shown as mean (standard deviation).

**Figure 2:**
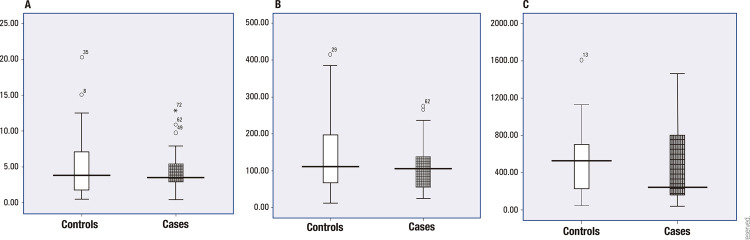
Box plots of (A) neutrophil-to-lymphocyte ratio (NLR), (B) platelet-to-lymphocyte ratio (PLR), and (C) platelet-to-monocyte ratio (PMR) in children with diabetic ketoacidosis (“cases”, patterned bars) and children with community-acquired pneumonia (“controls”, white bars).

The primary outcome of the study (*i.e.*, NLR) did not differ significantly between groups. The NLR among children with DKA and CAP had mean values of 4.77 (3.35) and 6.13 (9.36), respectively, and median values of 3.53 (IQR 2.87–5.32, minimum-maximum 0.39-12.97) and 3.91 (IQR 1.74-6.94, minimum-maximum 0.51-57.72), respectively (p = 0.10). Similarly, no significant difference was observed between the groups regarding hemoglobin level, red blood cell count, TLC, ANC, basophil count, monocyte count, or hematocrit values. However, significant differences were observed between groups regarding PLR, PMR, and eosinophil count. The mean PLR was 108.26 (67.51) in the case group and 166.60 (163.83) in the control group (p = 0.01), the mean PMR was 1,795.40 (4,307.0) and 886.33 (1,726.41), respectively (p = 0.01), and the mean eosinophil count was $0.56(1.41)\times 1,000/{mm}^{3}$ , and $0.29(0.80)\times 1,000/{mm}^{3}$ , respectively (p = 0.01). Figure 2 shows the distributions of NLR, PLR, and PMR in both groups.

Table 2 shows the analysis of the relationship between baseline hematological parameters and clinical characteristics and outcomes in children with DKA.

**Table 2:** Correlation matrix showing the relationship* between hematological parameters and outcomes in the group with diabetes ketoacidosis (n = 35)

Hematologic parameters	Outcome variables	DKA severity	Shock requiring vasopressors	Cerebral edema	Duration of hospital stay	Mortality
Total leukocyte count		0.30 (0.07)	-0.13 (0.42)	-0.16 (0.33)	-0.32 (0.06)	-0.11 (0.52)
Absolute neutrophil count		**0.37 (0.02)**	-0.08 (0.64)	-0.09 (0.57)	-0.29 (0.08)	-0.08 (0.61)
Lymphocyte count		-0.07 (0.68)	-0.24 (0.16)	-0.27 (0.10)	-0.28 (0.10)	-0.15 (0.37)
Neutrophil-to-lymphocyte ratio		0.25 (0.14)	0.31 (0.06)	0.27 (0.11)	0.03 (0.85)	0.07 (0.66)
Platelet count		0.04 (0.79)	-0.08 (0.63)	-0.15 (0.36)	-0.03 (0.85)	0.04 (0.80)
Platelet-to-lymphocyte ratio		0.11 (0.51)	0.20 (0.24)	0.22 (0.19)	0.28 (0.10)	0.13 (0.43)
Monocyte count		-0.17 (0.30)	-0.15 (0.36)	-0.27 (0.10)	0.08 (0.64)	-0.05 (0.77)
Platelet-to-monocyte count		0.25 (0.14)	**0.68 (0.00)**	**0.39 (0.01)**	-0.08 (0.62)	0.25 (0.14)
Eosinophil count		-0.18 (0.29)	-0.12 (0.49)	-0.18 (0.27)	0.18 (0.30)	-0.09 (0.58)
Basophil count		-0.16 (0.34)	-0.04 (0.81)	0.00 (0.97)	0.08 (0.63)	0.03 (0.84)
Hemoglobin		-0.28 (0.10)	-0.29 (0.08)	**-0.38 (0.02)**	-0.06 (0.69)	**-0.40 (0.01)**

*Values are shown as correlation coefficient (p value). Abbreviation: DKA, diabetic ketoacidosis.

Significant positive correlations were observed between ANC and DKA severity, PMR and shock, and PMR and cerebral edema, while significant negative correlations were observed between hemoglobin level and cerebral edema, and hemoglobin level and mortality.

## DISCUSSION

The results from this comparative cohort study indicate that children with DKA, compared with children with CAP, were significantly more likely to have abdominal pain, vomiting, and dehydration and less likely to have pallor at presentation. The duration of the symptoms was longer among children with DKA. The primary outcome of our study (NLR) did not differ significantly between the groups; however, significant differences were observed between groups regarding PLR, PMR, and eosinophil counts. Regarding the analysis of the relationship between baseline hematological and clinical characteristics and outcomes, significant positive correlations were observed between ANC and DKA severity, PMR and shock, and PMR and cerebral edema, while significant negative correlations were observed between hemoglobin level and cerebral edema, and hemoglobin level and mortality.

In our study, the average mean age of children with DKA was 7.4 (4.08) years; the youngest child was 1 year old and the oldest was 15 years old. Out of 35 children, 12 (35%) were male and 23 (65%) were female. A similar study was conducted by Bilici and cols. (2011) including 26 children with DKA (mean age 9.15 [3.85] years, range 4-15 years; 10 girls and 16 boys), of whom 58.3% had developed DKA for the first time and had the diagnosis of diabetes mellitus recently established (18). The period of prospective enrollment in our study coincided with the COVID-19 pandemic. Since the literature clearly indicates an effect of COVID-19 infection on hematological parameters of inflammation, the occurrence of COVID-19 infection was likely to confound our observations (6,7). Hence, all the children were enrolled after ruling out active COVID-19 infection, which was a standard procedure followed for all hospital admissions during that period.

Our study found that common clinical presentations in the case group were fever (34%), breathlessness (65%), vomiting (51%), abdominal pain (37%), hypoxia (40%), pallor (8.5%), and respiratory distress (51%). These findings were comparable to those reported by Kanwal and cols. (2012), who described polyuria and polydipsia (54.5%), persistent vomiting (52.7%), altered sensorium (50.9%), and abdominal pain (47.3%) as common symptoms at presentation among children with DKA (19). A retrospective Chinese study by Peng and cols. (2021) in 681 children diagnosed with T1DM found that 89% had polyuria and 91% had polydipsia, with children with DKA more likely to report vomiting, abdominal pain, and – particularly – fatigue. Their study reported a distribution of cases according to DKA severity comparable to that found in our study (20). Among children with DKA in the present study, the mean duration of hospital stay was 6.51 (2.21) days; among them, 8.5% required inotropic support, and 8.5% required support with mechanical ventilation. In a study by Singh and cols. (2019) in patients presenting with DKA, the mean duration of hospital stay was 8.2 (5.0) days, and low hemoglobin level (p = 0.019) and high pulse rate (p = 0.025) were independent predictors of longer hospital stay (21).

Our study found a 20% rate of cerebral edema and a 5.7% mortality rate. The rate of children with cerebral edema was higher than that reported by Patel and cols. (2016), who estimated an incidence of 0.39% in their study (22). Prior therapy with intravenous fluids could be the reason for a higher rate of cerebral edema. Glaser (2001) found that cerebral edema was the most frequent serious complication of DKA in children, occurring in 1%-5% of all DKA episodes, with high rates of mortality and permanent neurologic morbidity resulting from this complication (23).

The mean age of the children with CAP in our study was 7.9 (3.57) years. Common clinical presentations among children with CAP were fever (100%), breathlessness (30%), vomiting (2.5%), abdominal pain (10%), hypoxia (55%), pallor (20%), and respiratory distress (62.5%). Awasthi and cols. (2022) studied children (ages < 5 years) with CAP and observed that among 71.9% (7,196 of 10,006) of those with severe pneumonia, 35.9% (2,580 of 7,196) had hypoxic pneumonia (24).

Among the 40 children with CAP in our study, the pneumonia severity (as per WHO criteria) was graded nonsevere in 19 (47.5%), severe in 13 (32.5%), and very severe in 8 (20%). The mean duration of hospital stay in this group was 9.07 (7.77) days, and 7.5% of these children required inotropic support. The mortality rate in this group was 2.5%. Kapoor and cols. (2022) found a 9.4% mortality rate among children with CAP who were younger than 5 years (25); the mortality was higher among children younger than 12 months, those living in rural areas, and those without immunization or with partial immunization for their age.

Our study found comparable NLR values between groups, with mean NLR of 4.77 (3.35) and 6.13 (9.36) among children with DKA and CAP, respectively (p = 0.10). In contrast, significant differences were observed between groups regarding PLR, PMR, and eosinophil count. Salah and cols. (2022) reported greater NLR (p = 0.008) and lower PLR (p = 0.007) in children with diabetes-related microvascular complications compared with healthy controls (26). Multivariate logistic regression revealed that microvascular complications were independently associated with NLR (p = 0.013) and PLR (p = 0.004) (26). Demirtas and cols. (2015) observed that abnormal hematological indices were closely associated with glycated hemoglobin (HbA1c) levels in individuals with and without diabetes, and that these parameters were associated with diabetic microvascular complications (27). Hence, NLR and PLR could potentially be indicators of risk of development of diabetic microvascular complications in children with T1D. Kartal and cols. (2017) observed that NLR, PLR, and C-reactive protein (CRP) levels were significantly higher in individuals with CAP compared with controls (28). Liu and cols. (2016) analyzed 278 ICU admissions related to DKA, stratified by PLR cutoff value (29). They found that the incidence of readmission and mortality was 17.8% in the high PLR group, which was significantly higher than the 7.4% found in the low PLR group (29). Zheng and cols. observed significantly lower PLR values among children with bacterial pneumonia compared with children with mycoplasma pneumonia and healthy controls (p < 0.05) (30). Xu and cols. (2013) reported significantly higher total leukocyte count and neutrophil count but lower eosinophil count in cases with severe compared with mild/moderate DKA (p < 0.05) (31). Terai and cols. (2011) found that peripheral eosinophilia was characteristic in patients with acute pneumonia but not in those without lower respiratory infection (32).

Among children with DKA in our study, ANC and PMR correlated positively and hemoglobin level correlated negatively with unfavorable outcomes. Wang and cols. (2022) reported higher ANC values in patients with type 2 diabetes compared with individuals without diabetes ( $5.02[3.46]\times 10^{3}\text{ cells }/LL\text{ versus }4.47[3.66]\times 10^{3}\text{ cells }/\mu L$ , respectively, p = 0.004) (33). Christy and cols. (2014) found elevated HbA1c levels (6.8 [1.4]%) in individuals with diabetes and iron deficiency compared with controls, with levels even higher among women (7.02 [1.58]%) (34).

Our study was conducted at an exclusive pediatric teaching institute in India’s national capital region. This state-run institute caters to a wide spectrum of children from various sociodemographic backgrounds, coming from the adjoining north Indian region. The study involves the utilization of investigations that are inexpensive, widely available, and routinely performed as part of the standard care for children with DKA or CAP. The analysis was performed using statistical tools that are widely accepted for such data analysis. The data collection period coincided partially with the COVID-19 pandemic, which affected the behavior of patients seeking health care and, thus, their availability for enrollment in the study. The final sample reached in the case group (35 participants) was above the calculated sample size of 32 required to answer the study questions, with 80% power at a 90% confidence level. We consider that our study was reasonably successful in answering the key questions it was intended to answer.

The study has several strong points. It explored a widely recognized concept that had never been studied before. Leukopenia is a well-known occurrence that has been described for decades in the setting of pediatric DKA with or without evidence of an underlying infection. However, whether its characteristics differ from leukopenia observed in typical infections is a topic that has not been explored before. Further, the role of leukopenia as a predictor of clinical outcomes is a novel hypothesis. The study involves the use of inexpensive, widely available, and routinely performed laboratory parameters without posing any additional burden beyond the standard care. This makes the study easily replicable in any setting with or without sophisticated resources. The primary tools (*i.e.*, hematological parameters) are objective and free from any bias of subjective assessment. The limited follow-up duration, which included only the period of hospital admission, renders the reliability of the captured observation even stronger.

Despite thoughtful planning, the study has a few limitations; thus, the results must be interpreted with caution. The study was planned as a postgraduate thesis; hence, the period for sample collection was limited. Although we were able to reach the planned sample size in the control group, the sample size in the case group was only sufficient to interpret the results at a confidence level of 90% instead of 95%. Since our primary study tool (leukocyte response) is known to be affected by several physiological (age) and pathological (sepsis, hydration status, medication use) factors, the interpretation of our study findings may be complex. Although we deliberately explored inexpensive, routinely performed laboratory parameters for our study, the addition of sophisticated biomarkers like procalcitonin, high-sensitivity CRP, and cytokines, among others, would certainly add to the validity of the study results. Thus, further research with larger sample sizes and use of sophisticated tools and markers is suggested to explore these observations further in order to utilize them as a standard care in the management of children with DKA, especially in settings with limited resources.

In conclusion, the results of our study indicate significantly different characteristics of leukocytosis (PLR, PMR, and eosinophil count) in children with DKA compared with children with CAP. In addition, certain baseline hematological parameters (hemoglobin level, ANC, PMR) showed a significant correlation with disease characteristics (DKA severity) and outcomes (incidence of shock, cerebral edema, and mortality). These findings may be very relevant to regions with limited resources. A complete blood count – an inexpensive, widely available, and routine investigation performed at admission – may provide insight into some critical disease outcomes among children with DKA.

Authors contributions: Dr. Harish Kumar, Dr. Bhanu Kiran Bhakhri – substantial contributions to the conception or design of the work. Dr. Nupur Singh, Dr. Vernika Tyagi – acquisition, analysis, or interpretation of data for the work. Dr. Harish Kumar, Dr. Bhanu Kiran Bhakhri, Dr. Ruchi Rai – elaboration of the work or critical review of important intellectual content. Dr. Bhanu Kiran Bhakhri, Dr. Dharmender Kumar Singh, Dr. Ruchi Rai – final approval of the version for publication. Dr. Harish Kumar, Dr. Bhanu Kiran Bhakhri – consent to be responsible for all aspects of the work, ensuring that issues relating to the accuracy or integrity of any part of the work are properly investigated and resolved.
